# Chloral Hydrate as an Anticeptic

**Published:** 1878-05

**Authors:** 


Chloral Hydrate as an Antiseptic.—As an addendum to the abstract of Dr. Larrabee's paper in the last number of the *News* (p. 59), I desire to call attention to the special use of this agent as an application in uterine cancer. I have used it in a number of cases, and have found nothing equal to it. Not only does it correct the intolerable stench, but it relieves the stinging pains which are so common in these cases, and it changes the character of the discharges, making them more healthy, even at times almost resembling laudable pus. If by its use we can accomplish only the first of these objects, it is a sufficient warrant for a more extended trial of the remedy, and I feel confident that it will not disappoint expectation. I have employed it by saturating little pledgets of cotton with a solution of varying strength, according to the character of the discharge, of from ten to thirty grains to the ounce. By attaching a string to the pledgets they are easily removed by the patient after a few hours, without any danger of starting up hæmorrhage. The application is also of equal value on open cancers and foul sores of almost any character.

